# Multi-column modelling of lake Geneva for climate applications

**DOI:** 10.1038/s41598-021-04061-6

**Published:** 2022-01-10

**Authors:** Romain Gaillard, Marjorie Perroud, Stéphane Goyette, Jérôme Kasparian

**Affiliations:** 1 Institute for Environmental Sciences, University of Geneva, bd Carl Vogt 66, 1211 Geneva 4, Switzerland; 2 Group of Applied Physics, University of Geneva, Chemin de Pinchat 22, 1211 Geneva 4, Switzerland

**Keywords:** Climate change, Limnology

## Abstract

The interaction between large inland water bodies and the atmosphere impacts the evolution of regional weather and climate, which in turn affects the lake dynamics, thermodynamics, ice-formation, and, therefore, ecosystems. Over the last decades, various approaches have been used to model lake thermodynamics and dynamics in standalone mode or coupled to numerical atmospheric models. We assess a turbulence-closure $k-\epsilon$ multi-column lake model in standalone mode as a computationally-efficient alternative to a full three-dimensional hydrodynamic model in the case of Lake Geneva. While it struggles to reproduce some short-term features, the multi-column model reasonably reproduces the seasonal mean of the thermal horizontal and vertical structures governing heat and mass exchanges between the lake surface and the lower atmosphere (stratified period, thermocline depth, stability of the water column). As it requires typically two orders of magnitude less computational ressources, it may allow a two-way coupling with a RCM on timescales or spatial resolutions where full 3D lake models are too demanding.

## Introduction

Lakes influence the climate from the local to the synoptic scales. Their effects increase with their surface area, depth, and the magnitude of seasonal changes. They induce a thermal lag, affect heat and moisture budgets as well as wind speed and direction due to their low roughness height compared to surrounding land areas^1^.

Representing lakes in high-resolution numerical weather forecasting models^2^ and in regional climate models (RCMs)^3^ received much attention because it is of paramount importance to resolving fine-scale atmospheric processes in order to to reproduce weather and climate adequately. Efforts started few decades ago, when the atmospheric model surface grid spacing became fine enough to accommodate large freshwater bodies (e.g.^4–10^). However, lake-atmosphere coupling is still a challenge due to the complexity of interactions and feedbacks associated to local surface-atmosphere processes (e.g.^11^). Furthermore, the computing requirements of present 3D lake models are too intensive to realistically allow coupling them with climate models for durations above the year-range, especially in large lakes and at high spatial resolution^12^. Indeed, to date, long-term simulations of lakes properties at global or regional scales could mostly be performed with 1D models, e.g., within the ISIMIP consortium^13^. Considering the spatial variation implies considerable computational load and therefore constitutes a great challenge^14^.

Models showed genuine skill in reproducing many lake features based on different perspectives and simplifying assumptions. These include the eddy-diffusion approach^15–18^, the mixed-layer concept^5^, the Lagrangian approach (e.g. in the one-dimensional Dynamic Reservoir Simulation Model DYRESM^19^, where the thickness of each layer dynamically evolves over the course of the simulation, so that energy is redistributed across dynamic voxels), the *k*– turbulence method such as in Simstrat^20,21^, and self-similarity (assumed fixed shape) of the temperature-depth curve (Freshwater Lake model “FLake”^22^).

In order to resolve large lakes as lower boundary conditions in atmospheric numerical model in a realistic manner while limiting computing resources, a common practice consists in employing a single one-dimensional column model, or multiple independent columns taking into account the local lake bathymetry, depth, and surface forcing (*e*.*g*.^4^).

Such an approach offers wide perspectives for applications in weather prediction and climate simulations. In aquatic ecology, it may allow investigations into the relationships between physical, biological, and chemical quantities in large lakes over seasonal and inter-annual timescales. However, an intermediate option offering some insight into the horizontal and vertical thermal structure of the lake at a reasonable computing cost is desirable.

The goal of this study is to determine to which extent a multi-column lake model (MCM), *i*.*e*. 1D lake models distributed spatially across a 2D domain to form a quasi-3D representation of a lake, can offer such a computationally-efficient yet realistic option to represent the horizontal and vertical spatial thermal patterns of a large lake over seasonal time scales while avoiding the highly demanding computational requirements of a full 3D hydrodynamical model when used for long-term studies. As horizontal heat advection is disregarded in multi-column models, the assumption behind this work is that the overall heat transport across the water temperature profiles averages out to a certain extent over seasons.

More specifically, we focused on Lake Geneva, the largest and deepest alpine lake of Western Europe. By providing a high spatial heterogeneity Lake Geneva provides a stringent benchmark for a quasi-3D model. We compared simulated water temperatures from a MCM based on the 1D *k*–$\epsilon$ Simstrat lake model^21^, with those from the Delft3D full 3D hydrodynamical model^23^. Both models are driven by spatially-resolved surface forcing provided by simulated outputs from an atmospheric model. The reason for using the 3D model as a reference for the assessment of the MCM is twofold. On the one hand, we lack measurements with adequate temporal and spatial resolutions. On the other hand, in the prospect of substituting the MCM to the 3D model in climate studies, the practical question amounts to evaluating the impact of this substitution on the simulation quality.

We find that seasonal averages of the horizontal structures and vertical profiles of temperature simulated by the MCM and Delft3D are comparable, including during the stratification and de-stratification periods. The MCM therefore provides a useful alternative modelling tool for climate studies, although it does not capture short-term features like transient lake mixing events following strong wind episodes that would be required for regional weather forecasting. As it runs at least two orders of magnitude faster than its 3D counterpart, it can efficiently be nested in a RCM or allow multiple realizations of simulations with different initial conditions, allowing for statistical assessments or variability analyses and providing valuable spatial information while keeping an affordable computational cost.

**Figure 1:**
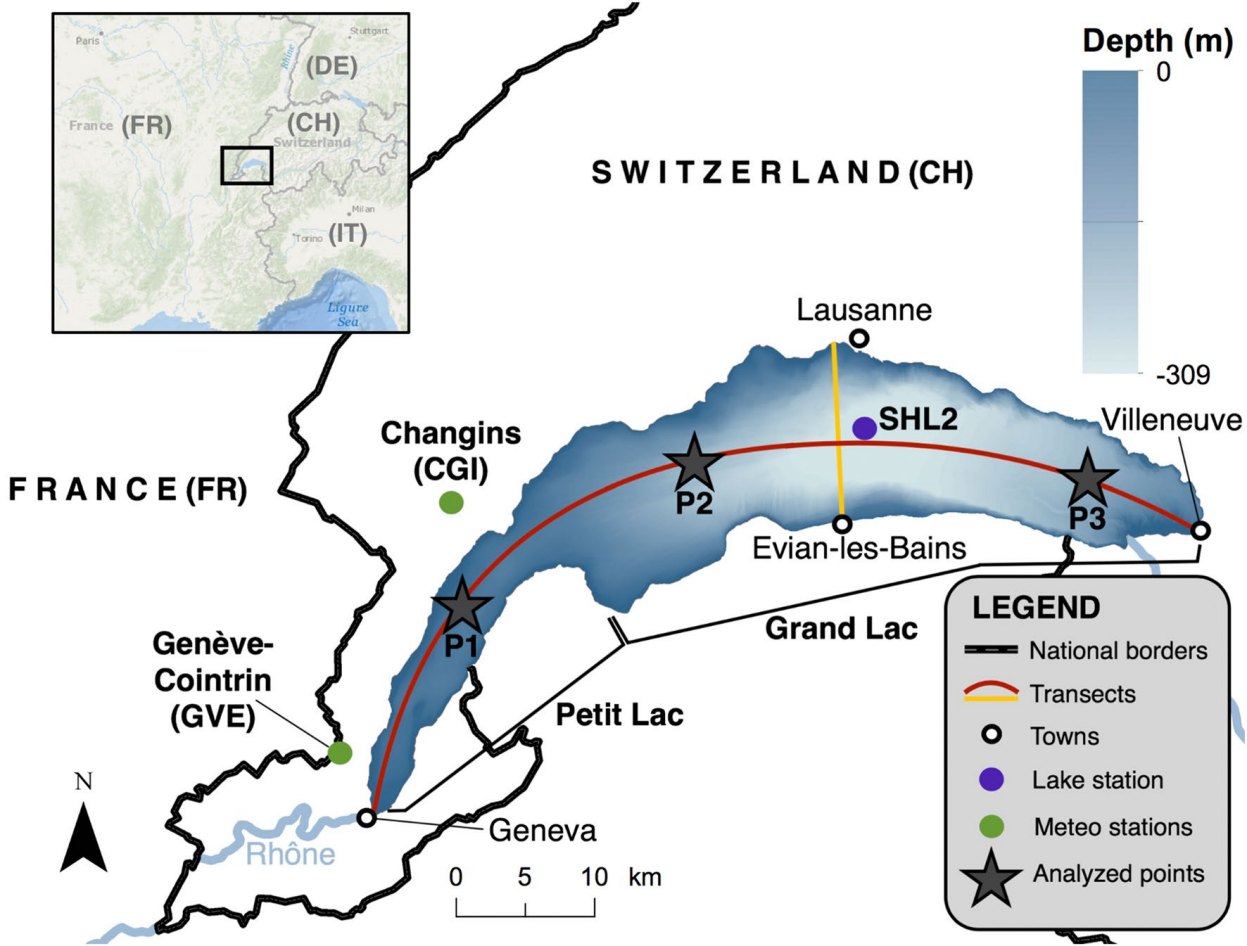
Study site. The yellow and red curves correspond to the N–S and W–E transects, control point P1 is located in the “Petit Lac”, whereas points P2 and P3 are located in the “Grand Lac”. Coordinates for upper right corner: 46.76° N/6.95° E and for lower left corner: 46.12° N/5.90° E. Map generated with ArcGis^24^.

## Results and discussion

**Figure 2:**
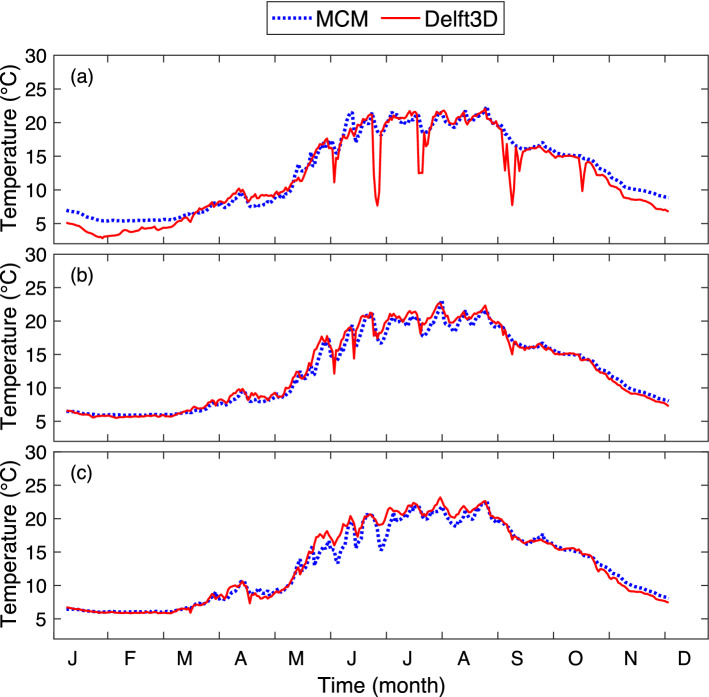
Surface water temperature from the MCM and Delft3D at points (**a**) P1, (**b**) P2, and (**c**) P3 (See map in Fig. 1). Plots generated with Matlab^25^; Panels assembled with Adobe Illustrator^26^.

**Figure 3:**
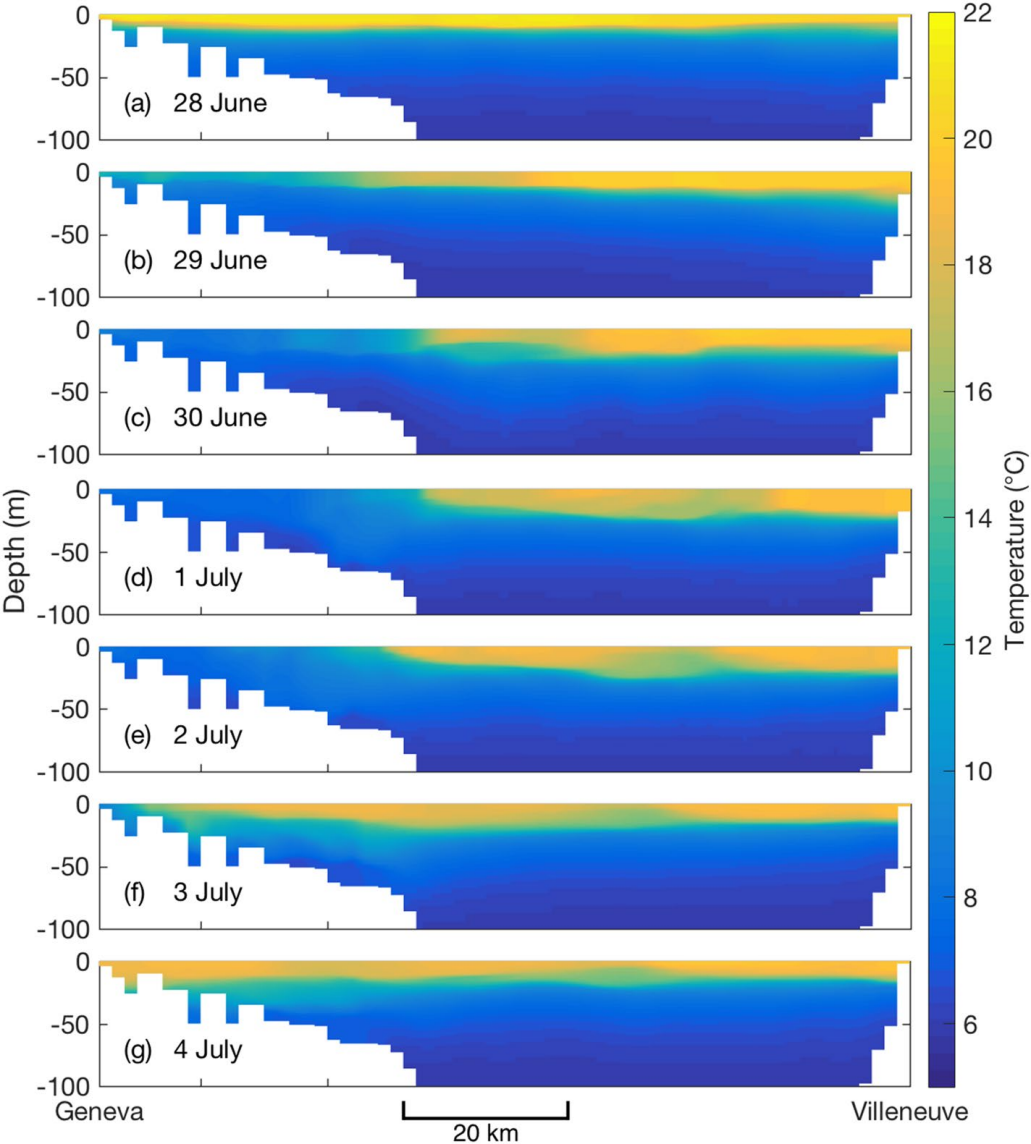
Upwelling event on June 28–July 4. Temperature profiles on the W–E transect at 1100UTC as simulated by Delft3D. Plots generated with Matlab^25^; Panels assembled with Adobe Illustrator^26^.

Figure 2 displays the evolution of the surface temperature of Lake Geneva at points P1, P2, and P3 (shown on the map in Fig. 1) as simulated by the MCM and Delft3D (See “Methods” for details). The overall trends are similar in both models, with few exceptions. At point P3 a mixing event due to strong wind (12 m/s) around 24–25 May is overestimated by the MCM, resulting in a drop in temperature as compared to Delft3D. Furthermore, during several short-time events the temperature predicted by Delft3D drops by up to 7 °C to 10 °C for periods of typically 4 days at point P1 (in the Petit-Lac, Panel a). During these upwelling events the warm surface layer of the western part of the lake, which is located upwind, is pushed eastward by the wind and replaced by colder water from deeper layers, especially along the coasts (compare Fig. 3c–e with Fig. 3a,g; Fig. 3b,f providing intermediate steps). As it is induced by horizontal dynamics, this process cannot, by essence, be reproduced by the MCM. Daily surface maps from Delft3D show that this is particularly the case close to the lake shore, which is also more favorable to the narrower part of the lake.

**Figure 4:**
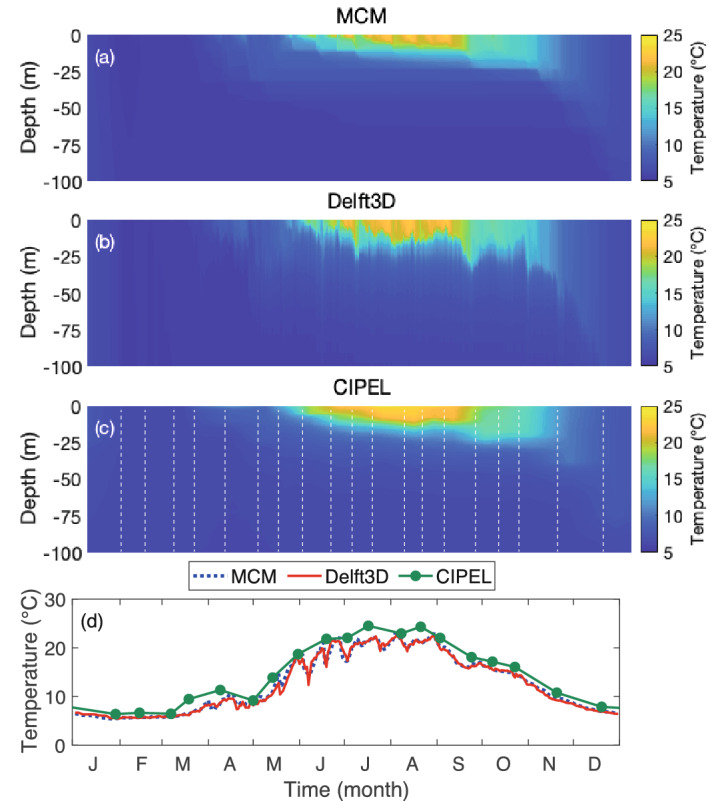
Evolution of the water temperature at 1100 UTC at station SHL2. (**a**–**c**) Vertical profiles from (**a**) the MCM, (**b**) Delft3D, and (**c**) CIPEL measurements. (**d**) Bulk surface temperature. Plots generated with Matlab^25^; Panels assembled with Adobe Illustrator^26^.

The temporal evolution of the temperature profiles over the year 2017 at point SHL2 (Fig. 4a–c) display comparable stratification periods (April–November) and thermocline depths in both models, as well as in the measurements. The MCM however predicts a slightly thinner epilimnion in Summer, and a smoother lake internal dynamics related to the lack of horizontal advection as well as to other unresolved processes, while Delft3D simulations are more noisy. Furthermore, both models underestimate the surface temperature in Summer (Fig. 4d), suggesting that they overestimate the vertical heat diffusion. Note that, since the columns of the MCM are independent from each other, and since SHL2 is the deepest point of the lake, the MCM outcome at this location is equivalent to that of a 1D model.

**Figure 5:**
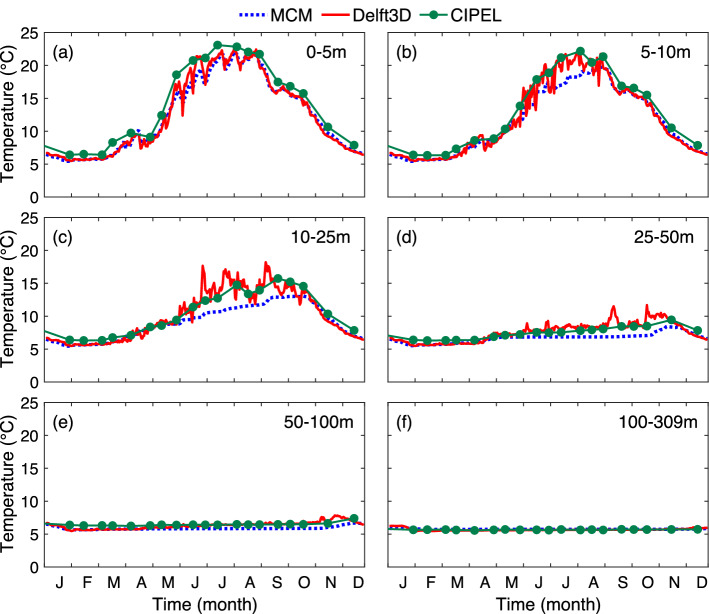
Evolution of the temperature simulated by the MCM and Delft3D at 1100UTC at station SHL2 at (**a**) the surface (0–5 m), (**b**) 5–10 m, (**c**) 10–25 m, (**d**) 25–50 m, (**e**) 50–100 m, (**f**) 100-309 m. Plots generated with Matlab^25^; Panels assembled with Adobe Illustrator^26^.

More details can be brought about by looking at each depth range (Fig. 5). The peak temperature is reached later at deeper layers, related to the time needed by the vertical transport to diffuse heat downwards. The MCM, that underestimates this transport, predicts cooler and later temperature maxima especially in intermediate layers. Furthermore, close to the surface (0–5 m, Fig. 5a), Delft3D predicts more marked fast temperature oscillations. However, beyond these discrepancies, both models evolve consistently with each other (0.6 °C RMS at SHL2 in that layer), as well as with the CIPEL measurements (0.96 °C and 0.3 °C RMS for the MCM and Delft3D, respectively). Note that, even at this location where it was specifically calibrated, the 1D standard, bassin-wide Simstrat model performs only slightly better than its multicolumn counterpart (0.43 °C with regard to the CIPEL measurements), without offering the horizontal resolution of the MCM and the associated variability over the whole lake.

The fast oscillations simulated by Delft3D dampen with depth down to 25 m depth (Fig. 5b,c). The temperature trends between 5 and 25 m are similar in both models although the MCM underestimates the temperature rise by up to 2.5 °C during the stratified period. This large temperature discrepancy at intermediate depths is the main limitation of the MCM. However, it can be understood as stemming from the combination of a strong vertical thermal gradient and a vertical offset of the thermal profile, especially in spring. The agreement is better ($|\Delta T| \le {1}\,^\circ {\text{C}}$) outside the stratified period. This temperature difference persists down to 100 m, although it damps with depth (Fig. 5d,e). The underestimation of the temperature rise in the metalimnion during Summer is typical of a less efficient heat transport to the metalimnion as the 3D dynamics is not included in spite of the parametrization of the vertical diffusion. However, the temperature difference between the two models vanishes in Autumn, showing that the MCM also underestimates the upward heat flux, limiting the temperature drop in the metalimnion in the MCM.

Finally, the temperature below 50m (Fig. 5e,f) is highly stable over the year and very similar in the MCM and in the Delft3D.

**Figure 6:**
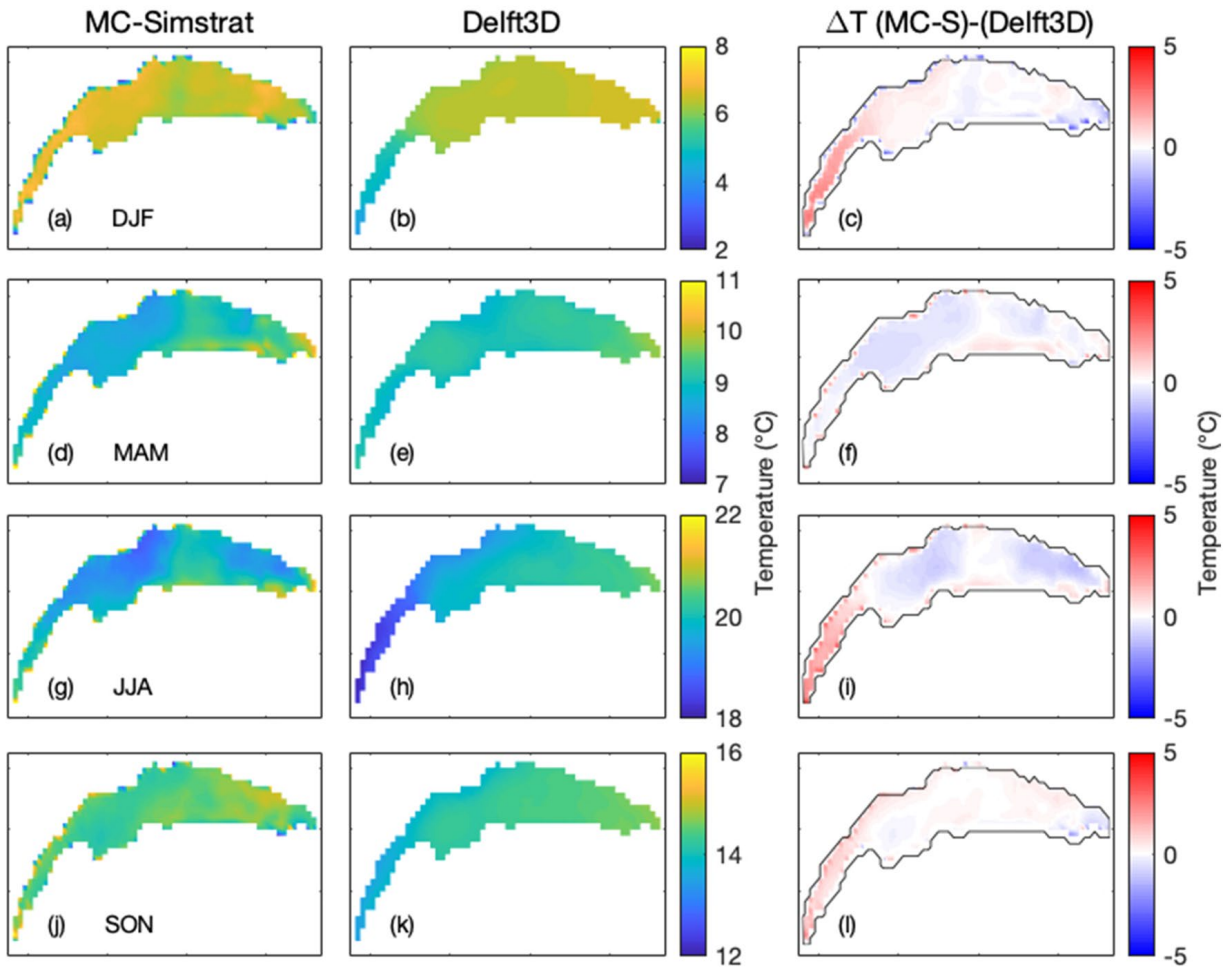
Seasonally-averaged surface (0 m) temperature maps from the MCM (col 1), Delft3D (col 2), and their difference (MCM—Delft3D) (col 3). (**a**–**c**) Winter (DJF), (**d**–**f**) Spring (MAM), (**g**–**i**) Summer (JJA), (**j**–**l**) Autumn (SON). Lake contours are indicated in Col. 3 for clarity. Plots generated with Matlab^25^; Panels assembled with Adobe Illustrator^26^.

Figure 6 compares the seasonally-averaged lake surface temperature maps from both the MCM and Delft3D. Differences are limited to $|\Delta T| \le {1}\,^\circ {\text{C}}$ over most of the surface. A notable exception occurs in the Petit Lac, where the MCM overestimates the temperature by up to 4.6 °C as compared to Delft3D, except in Spring. This discrepancy can be attributed to the less efficient heat transport down to the metalimnion as described earlier, especially during upwelling events. The heat outflow at the western end of the Petit Lac into the Rhône river, that is taken into account in Delft3D and neglected in the MCM, is also likely to contribute. With its higher resolution and three-dimensional dynamics, Delft3D also resolves mixing more accurately, especially during the transient partial mixing events. These events cool down and homogenize the surface temperature, especially in the center and eastern side of the lake (Fig. 6).

**Figure 7:**
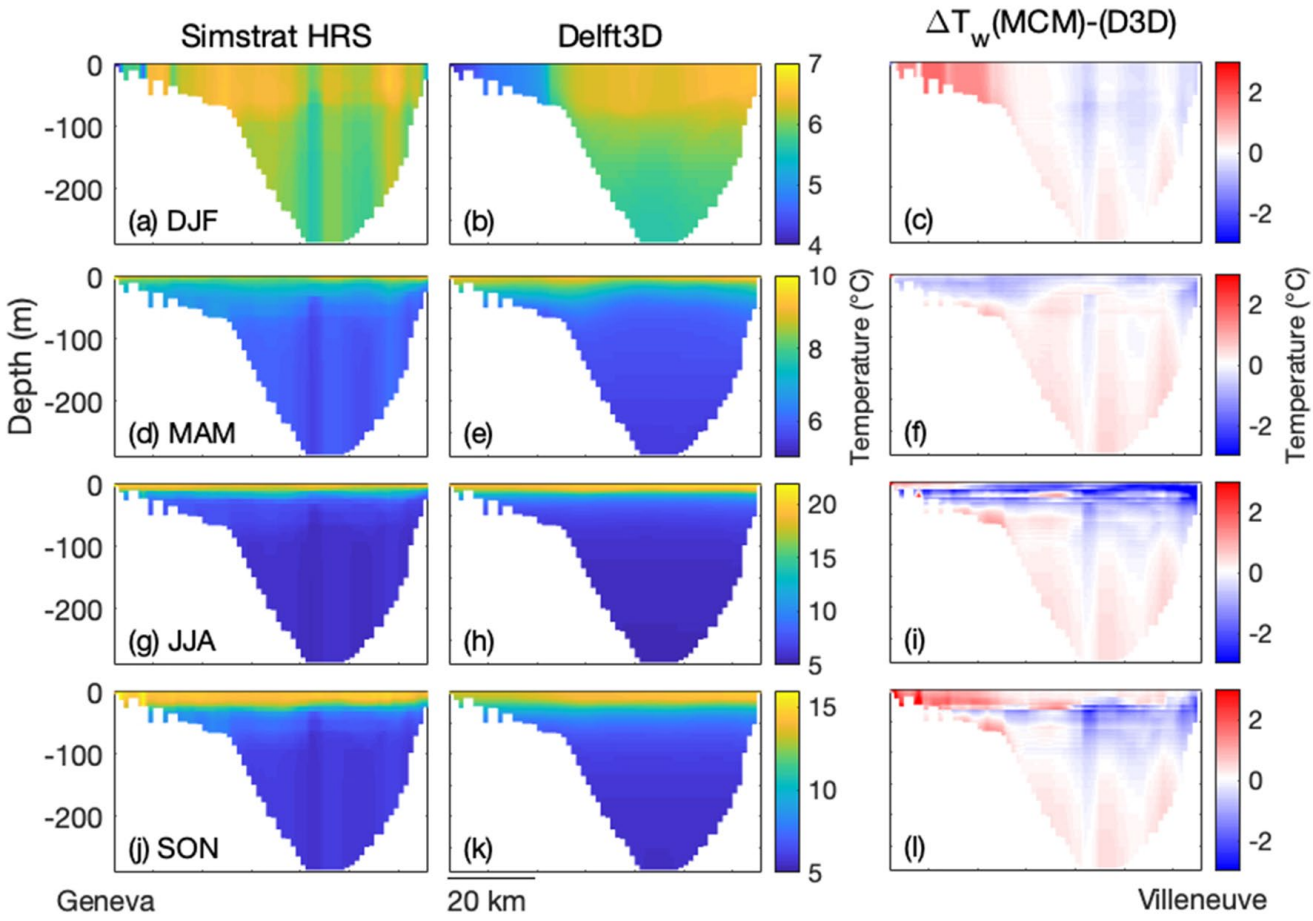
Season-averaged W–E transects (See Fig. 1) for the MCM (col 1), Delft3D (col 2), and the difference MCM–Delft3D (col 3). (**a**–**c**) Winter (DJF), (**d**–**f**) Spring (MAM), (**g**–**i**) Summer (JJA), (**j**–**l**) Autumn (SON). See Fig. S2 for monthly details. Plots generated with Matlab^25^; Panels assembled with Adobe Illustrator^26^.

Seasonally-averaged W–E transects (Fig. 7) obtained from both the MCM and Delft3D are quite similar. In particular, they display stratification at comparable depths, with 8.8 m RMS difference between the two models. However, this slight shift, combined with strong vertical temperature gradients up to 2.1 °C/m at the thermocline, lead to a temperature difference reaching up to 4.9 °C in this layer appearing as sub-surface blue bands on the last column of Fig. 7. The highly localized cold column predicted by the MCM in the middle of the transect along the longer lake axis, especially in Winter and Spring (See first column) may be due to inhomogeneities in the wind forcing, reinforced by the fact that the MCM does not explicitly resolve horizontal mixing, allowing gradients to develop and persist, as evidenced by the rise of temperature contrasts during the spinup.

Similar results are noticed for the N–S transect (Fig. S1). The monthly-averaged data (Figs. S2 and S3) show that the lake tends to stratify (resp. destratify) earlier in Delft3D, as evidenced by the warmer (colder) epilimnion in March–April (November), in particular on the N–S transect (Fig. S3g,h, j,k,ah,ai). This appears as a negative blue (resp. positive red) layer in the temperature differences of Fig. S3i,l (resp. Fig. S3aj).

**Figure 8:**
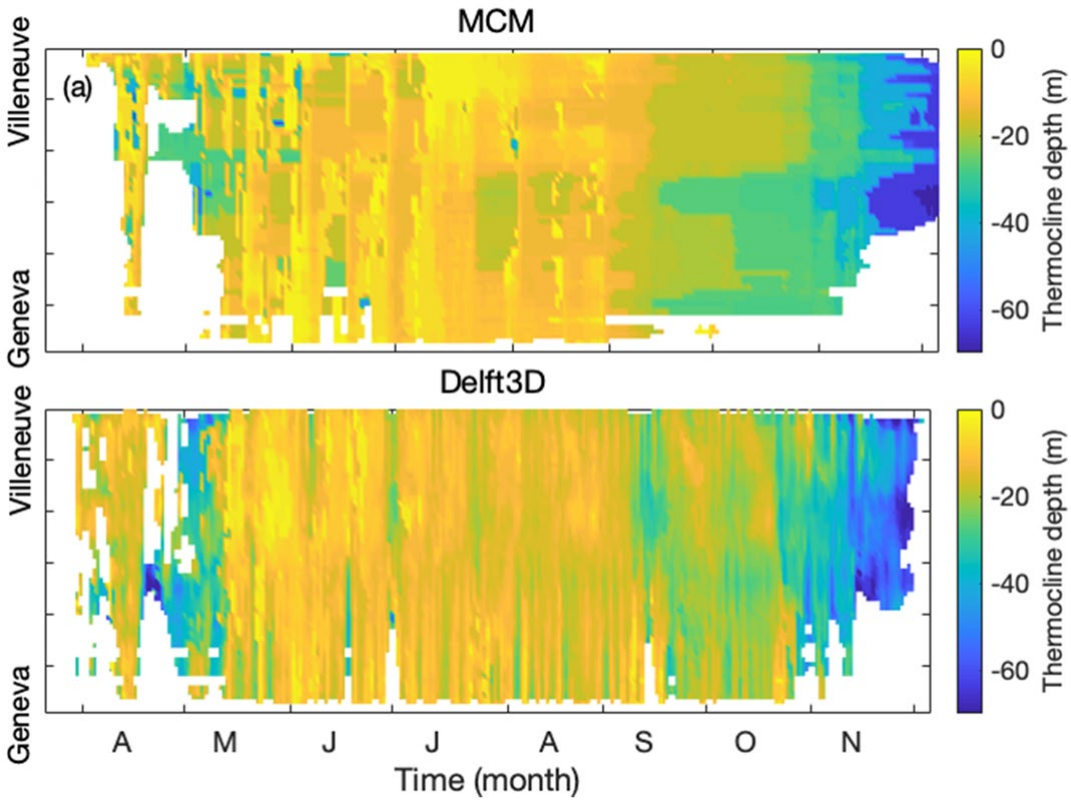
Temporal evolution of the thermocline depth over the the W–E transect, as simulated by (**a**) the MCM and (**b**) Delft3D. Plots generated with Matlab^25^; Panels assembled with Adobe Illustrator^26^.

**Figure 9:**
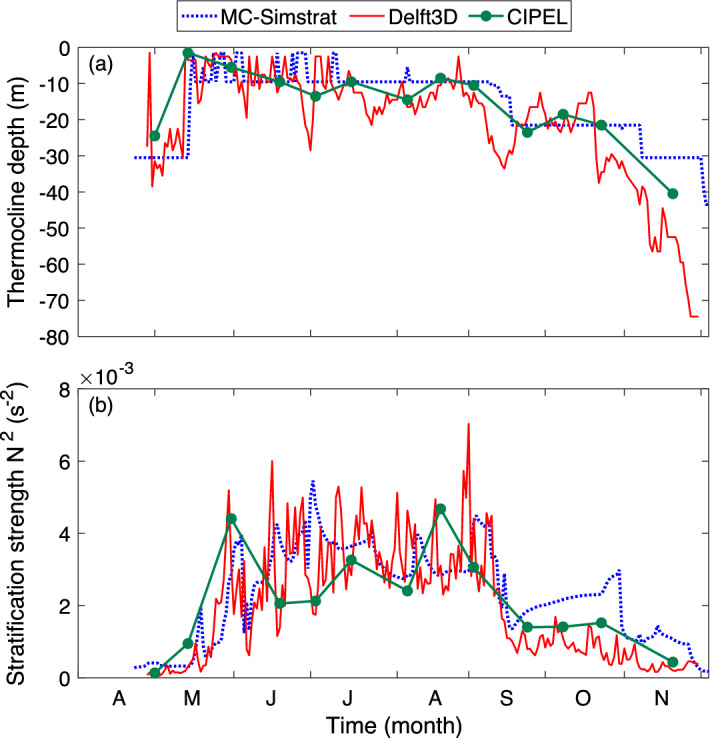
Temporal evolution of the simulated and measured (**a**) thermocline depth and (**b**) stability index $N^2$ at point SHL2. Plots generated with Matlab^25^; Panels assembled with Adobe Illustrator^26^.

During the stratified period (June to October), both model predict similar thermocline depths and temporal evolutions (Fig. 8), with a RMS difference of 7m on both transects. Differences are slightly larger during stratification (April, May) and destratification (November), with, e.g., RMS differences of 11 m on the W–E transect during both periods. This is exemplified in Fig. 9 in the case of point SHL2.

Due to the less efficient vertical heat exchanges in the MCM, the stratification once occurring is more steady. Therefore, unlike for the appearance of the first stratification, the *established* stratification (i.e., stratification maintained for at least 30 days) is reached only 7 days earlier in the MCM (April 23 *vs* April 30) and lost almost simultaneously in the two models (December 4 and 5, respectively). A lag is however noticed in the Petit lac, that is fully destratified as early as mid-November in Delft3D, and only 18 days later (early December) for the MCM.

During destratification, the thermocline is simulated deeper by Delft3D (Figs. 8 and 9a), presumably due to the 3D dynamics. Note that the CIPEL measurements lie between the two model outputs. Finally, the column thermal stability index $N^2$ at the thermocline (Fig. 9b) is similar in both models during this period, whereby the MCM underestimates the column stability by only 6% between May and August. However, the shallower thermocline during the destratification in the MCM is accompanied by a much stabler column in September and October. Again, similar results are obtained on the N–S transects (Fig. S4). Note that these discrepancies occur at the end of the stratified season, when the stratification is weaker and therefore has a more limited effect on the surface temperature maps, hence on the heat exchanges with the atmosphere.

The thermocline depth and the thermal stability index have larger short-term fluctuations in Delft3D, where mechanical turbulence due to shear stress induces small-scale turbulent motions that translate into local inhomogeneities of both the thermocline and the water column stability.

Both the MCM and Delft3D lake models predict comparable seasonal means of the spatial surface temperatures patterns and a similar evolution of the water column temperature in spite of a lower temperature increase in the metalimnion in Summer in the MCM. In particular, the latter reproduces well the duration of the stratified period as well as the stability of the water column. These features must be reproduced prior to interfacing lake and atmospheric models, as they govern the thermal, momentum, and water vapour exchanges between the lake surface and the atmospheric boundary layer. A MCM lake model may therefore provide relevant input in view of a two-way coupling with a RCM for climate applications on timescales where full 3D lake models are too computationally demanding.

The surface (bulk, i.e. 0–1 m) temperatures from the MCM and Delft3D on long time scales are very comparable, with only 0.04 °C RMS. Fig. 6). The MCM however yields a colder temperature in Summer in the metalimnion (Fig. 5). This is consistent with a less efficient heat transport in the MCM, as the three-dimensional dynamics, is not considered. However, as this underestimation averages out over the year, it should still be compatible with climate modelling applications.

The main limitation of the MCM is its underestimation of the temperature at intermediate depths during the stratified period. Parametrizing the wind drag coefficient $C_\text {D}$ as in Eq. (2) instead of Eq. (1) allows Simstrat to better reproduce the Delft3D results at intermediate depths, ensuring adequate performance in the season with high heat exchange. This is especially the case for the thermocline depth as well as the temperature in the metalimnion (compare panels c–e in Figs. 5 and S5), thus showing the sensitivity to the parametrization of $C_\text {D}$. The larger drag coefficient of Eq. (2) enhances the energy transfer from the surface to deeper layers, as the seiching parametrization does. Due to the steeper vertical temperature gradient around the thermocline, the intermediate depths are the most sensitive to the parametrization of the drag coefficient. Note that the drag coefficient used by Delt3D for low wind speeds is 2 to 6 time higher than that of the MCM. It is remarkable that the MCM reasonably reproduces reasonable thermal profiles without requiring such high values of $C_\text {D}$.

Over shorter time scales, the MCM however proved unsatisfying to capture the full dynamics as well as transient effects implying three-dimensional heat exchange between adjacent columns in the lake. Accounting for these phenomena in a multicolumn model like the MCM would require parametrizations to emulate horizontal advection and other heat transport processes, such as the application of horizontal filters as described in^27^.

In view of coupling a lake model with a high resolution RCM, the computing requirements are crucial, as both high temporal and spatial resolutions are required. Typical model timesteps are tens of minutes for GCMs and minutes for RCMs. Furthemrore, spatial resolutions in the km-range correspond to over 500 grid points on Lake Geneva and several thousands of points for all Western-European lakes, e.g.^28^. In this regard, the use of a MCM brings a speed-up factor by at least two orders of magnitude while offering a reasonable representation of the spatial patterns of temperature. Simulating one year requires less than 25 min calculation on a single processor and a few GB of RAM with the MCM, as compared with 2–7 days on 2 processors and 256 GB RAM with Delft3D. These values would scale to 0.5–2 days if using the same horizontal resolution in Delft3D as the MCM. Further speed-up may even be obtained with parallel computing: its implementation is straightforward given the independence of the water columns in a MC model. This would allow performing high-resolution multi-decennial simulations, as well as running multiple realizations to gather statistics with random initial conditions, perform sensitivity analyses, and/or investigate alternative scenarios. In this regard, the fact that the MCM remains stable after the 14-year spinup period suggests that it can stably perform long-term simulations, although the present study did not formally asses this ability. However, we note that MCM stability over the whole RCM simulation period is not critical, since the latter may drive the MCM in case of biases over multi-decennial time scales. We also note that the expected future increase in computing power may appear to reduce the interest for MCM. However, depending on their actual needs, future modelers may want to use the increased computing power to improve the horizontal or vertical resolution (e.g., to couple with a higher-resolution regional climate model) as allowed by the MCM, rather than to model in more details the physical processes by implementing a full 3D model.

Climate change may affect the lake thermal properties, as well as mixing regime^29^. The robustness of the multi-column model to changes in wind regime could be reduced: Indeed, the MCM proved to be less sensitive to upwelling events than Delft3D. Therefore, a change in wind regime^30^ that is expected to affect mostly extremes, might be detrimental to the MCM. On a monthly or seasonal time scale, the MCM should however still be able to mimic changes in mixing regime. Confidence is gained by the ability of the model to reproduce the thermal properties, the thermocline depth, the destratification period all over the year, independently of the lake depth. Although confidence in future changes in mean windiness still remains uncertain^31,32^, the MCM performed sufficiently well over each segment of the lake to believe it can be applied to other alpine lakes, providing reasonable spatially-resolved temperature patterns at an affordable computing cost.

## Conclusion

Resolving lakes as lower boundary conditions in high resolution numerical models for weather and climate represents more than a mere technical challenge. The choice of a particular lake model must be based on its skill to reproduce important lake characteristics as well as on the optimization of the computational load they require. While 3D hydrodynamical lake models coupled with a RCM can simulate the surface temperature distribution thus allowing realistic surface sensible heat and evaporation fluxes^12^, their computational resource requirements prevents long-term simulations. In this paper, we compared a multi-column and a 3D hydrodymanical lake models using the same boundary and initial conditions applied to Lake Geneva over the year 2017 in stand-alone mode. We showed that the seasonnally-averaged lake surface temperature pattterns are reproduced with reasonable accuracy over Lake Geneva by the multi-column approach. The implementation of such a model in a RCM would therefore allow to reproduce the local climate. As the thermocline and other lake thermodynamic features are reproduced by the MCM with a similar accuracy as Delft3D on a seasonally average basis, the approach described in this paper may therefore be appropriate to drastically reduce the computational cost of long-term simulations. While the long-term stability of the MCM has not been assessed in this work, the RCM can be used to reset the MCM border conditions in the case of possible multi-decennial drifts. In contrast, the multicolumn model behaves less accurately at the monthly or shorter timescales, which limits its relevance, at least in the present state, for weather forecasting. Simulations over other lakes and lake types, under various climate conditions, and over longer periods using the same multi-column lake-modelling approach, will assess the generality of our finding in the future.

## Methods

### Study site

Lake Geneva is the deepest (309 m) and largest (580 ${\hbox {km}^{2}}$) lake of Western Europe. It is surrounded by the Alps on its Southern shores and the Jura mountains on the Northern shores, and partitioned in two basins: a deep one (“Grand Lac”) on the Eastern side, and a shallow one (“Petit Lac”, 73 m) on the Western side (Fig. 1). Lake Geneva can be considered as a warm meromictic lake as it rarely overturns beyond 100–150 m depth^33^. Indeed, the eight latest mixing events occurred in 1970, 1971, 1981, 1986, 1999, 2005, 2006, and 2012^34^. However, the Petit Lac experiences full mixing each year.

We compared simulated outputs with observed water temperature profiles using 0.2 m to 0.5 m vertical spacings sampled by OLA-IS, AnaEE-France, INRAE of Thonon-les-Bains, CIPEL^35,36^ at the deepest point of the lake (station SHL2, see Fig. 1). These data are collected at least twice a month from Spring to Autumn, and at a lower frequency during Winter.

### Lake models

The multi-column modelling for the evolution of the lake thermal profile is based on the buoyancy-extended $k-\epsilon$ model^20^ Simstrat^21^. Turbulent mixing is computed from the turbulent kinetic energy (TKE) *k* and its dissipation $\epsilon$. Among forty-six alpine lakes, Simstrat exhibited a volume-weighted post-calibration root mean square error (RMSE) $< {1}\,^\circ {\text{C}}$ for 17 lakes, between 1 and 1.5 °C for 15 lakes, between 1.5 and 2 °C for 8 lakes and $>{2}\,^\circ {\text{C}}$ for only 6 lakes^37^.

The MCM features an independent flat-bottom column at each point of a two-dimensional gridded domain. Neglecting horizontal heat and mass transport allows for efficient computation while providing a first level of spatially-resolved modelling. The MCM lake model is driven by atmospheric meteorological forcing at a hourly time step. Simstrat implements an option to represent the vertical diffusion caused by the seiches. This parametrization depends on the depth and volume of the lake, and can be calibrated by two parameters. In the present study, these parameters have been calibrated using similar values for each column. The lake is considered as a closed basin, without water inputs and outputs flows. Lake ice and mass balance were not considered in this study.

The Delft3D flexible mesh suite^23^, hereafter denoted Delft3D, is a modular open source software simulating the hydrology of rivers, deltas and lakes. Beyond the processes taken into account in Simstrat, it implements in 3D the equations of continuity, momentum, and heat transport. Delft3D was run continuously since 2010 for Lake Geneva. It realistically replicated Lake Geneva’s thermal behavior and dynamics^38^. It provided lake surface temperatures and water temperature profiles every 3 hours via the Meteolakes plateform^39^.

### Input data and model parameters

Both models were forced with high spatial resolution, by the 1.1-km resolution version of COSMO (COSMO-1) providing hourly meteorological inputs. The driving variables include the surface downward shortwave ($R_s^\downarrow$) and longwave ($R_L^\downarrow$) radiation fluxes, the surface air relative humidity (RH) and screen-level air temperature *T*, thus yielding the partial vapor pressure *e*, and the anemometer-level wind speed *V* and direction, from which the eastward *u* and northward *v* wind components were derived. These values were linearly interpolated to the respective time steps of the models. *T*, *V*, and *e* were used to compute the surface sensible heat and evaporation fluxes, whereas *u* and *v* were used to compute the surface wind stress vector components.

Unless otherwise specified, the wind stress was parameterised in the MCM using a wind-dependent surface drag coefficient $C_\text {D}$^40–42^:

1a $$V \le 3\;{\text{m}}/{\text{s}}\quad C_{{\text{D}}} = 0.0044V^{{ - 1.15}}$$

1b $$3{\text{ < }}V \le 10\;{\text{m}}/{\text{s}} \quad C_{{\text{D}}} = \frac{{1.052 + 0.096V}}{{1000}}$$

1c $$V > 10\,{\text{m}}/{\text{s}}\quad C_{{\text{D}}} = 0.0013$$

In order to assess the sensitivity of the MCM to the drag coefficient and to get closer to the values used by Delft3D, we also performed a test with following alternative parametrization:

2a $$\begin{aligned}&V \le {10}\,{\text{m}/\text{s}}&C_\text {D}=0.0044 V^{-1.15} + 0.003 \end{aligned}$$

2b $$\begin{aligned}&V > {10}\,{\text{m}/\text{s}}&C_\text {D}=0.0013 \end{aligned}$$

while in Delft3D, the drag coefficient was the following^23^

3a $$\begin{aligned}&V < {0.5}\,{\text{m}/\text{s}}&C_\text {D}=0.0421 \end{aligned}$$

3b $$\begin{aligned}&0.5 \le V < {5}\,{\text{m}/\text{s}}&C_\text {D}=0.0467-0.0091V \end{aligned}$$

3c $$\begin{aligned}&5 \le V < {25}\,{\text{m}/\text{s}}&C_\text {D}=0.0009+5\times 10^{-5}V \end{aligned}$$

3d $$\begin{aligned}&V \ge {25}\,{\text{m}/\text{s}}&C_\text {D}=0.0021 \end{aligned}$$

A constant air density of 1.24 ${\hbox {kg/m}^{3}}$ was considered in both models. The absorption coefficient $K_\text {e}$ was deduced from the measured Secchi disk depth $Z_\text {SD}$^43^

4 $$\begin{aligned} K_\text {e}=-\frac{\ln (100)}{Z_\text {SD}}. \end{aligned}$$

Finally, in both models the surface water albedo was assumed constant at $\alpha$ = 0.09 and the thermal infrared emissivity fixed at 0.985^38^.

We relied on the bathymetry derived from the digital elevation model DHM25 of the Swiss Federal Office of Cartography Swisstopo^44^. Delft3D was run on 100 unevenly-distributed levels, with a higher resolution near the surface. The MCM featured a fixed 1 m vertical grid spacing. Although Simstrat has already been run with vertical resolutions as fine as 0.25 m^45^, such a resolution is beyond the needs of the present study, as it largely exceeds that of our reference, Delft3D. Furthermore, very high resolutions may result in numerical instabilities^46^.

Delft3D was run with a 400 m grid spacing implying a surface horizontal interpolation of the COSMO-1 driving data. In contrast, as the columns are independent in the MCM, we centered its 542 columns of $1.1 \times {1.1}\,{\hbox {km}^{2}}$ on the COSMO-1 grid mesh and used the bathymetry and depth of the nearest Delft3D mesh point to optimize the comparison.

The archival frequency of lake-model outputs was daily at 1100 UTC in order to allow comparison with observational records usually made around this time.

The analysis was performed on the first full year of COSMO-1 data availability, *i*.*e*., 2017. A fourteen-year spin-up of the MCM was necessary to reach a steady-state. During the spin-up, the temperature at the surface and below 50 m rose by 0.4 °C and $\lesssim {1}\,^\circ {\text{C}}$, respectively. After the spinup period, the multicolumn model was run over one year and its output was compared with Delft3D simulated thermal profiles.

### Model setup and post-processing

We checked that neither the albedo nor the value of surface water infrared emissivity values significantly impact the output of the MCM. Replacing a constant albedo by a parametrization as $\alpha =0.05/\left( \cos \theta _z +0.15\right)$^47^, $\theta _z$ being the local solar zenith angle, affects the output surface temperature by no more than 0.07 °C RMS. Reducing the thermal infrared emissivity from 0.985 to 0.97 changes the surface temperature by only 0.12 °C RMS over the year, and by ≈ 0.01 °C in deeper layers.

The vertical stability analysis was based on the vertical density gradient deduced from the vertical temperature profile output from each model. A thermocline was considered to settle if the vertical density gradient of water is sufficient, *i*.*e*. $\rho (z={100}{\text{m}}) - \rho (z={2}{\text{m}}) \ge {0.1}\,{\hbox {kg/m}^{3}}$, with *z* the vertical coordinate. We considered that stratification was established if this condition was fulfilled over at least 30 successive days. In this case, the thermocline depth was chosen to the maximum of the vertical density gradient, smoothed with a moving average over 10 m. We quantified the stratification strength via the water column thermal stability index $N^2$ at the maximum value,

5 $$\begin{aligned} N^2=- \frac{g}{\rho } \frac{\partial \rho }{\partial z}, \end{aligned}$$

where *N* is the buoyancy frequency and *g* is the gravitational acceleration.

## Supplementary Information

Supplementary Information 1.
