# Supplementary material for: Multi-column modelling of lake Geneva for climate applications
Source: Sci Rep. 2022 Jan 10;12:353. doi: 10.1038/s41598-021-04061-6 (PMC8748647; doi:10.1038/s41598-021-04061-6)
Supplement: Supplementary file 1 — Supplementary Information 1. [file 41598_2021_4061_MOESM1_ESM.pdf]

Multi-column modelling of lake Geneva for climate applications
Supplementary figures

Romain Gaillard[1,2], Marjorie Perroud[1], Stéphane Goyette[1,2], and Jérôme Kasparian[1,2,*]

[1]Institute for Environmental Sciences, University of Geneva, bd Carl Vogt 66, 1211 Geneva 4, Switzerland
[2]Group of Applied Physics, University of Geneva, Chemin de Pinchat 22, 1211 Geneva 4, Switzerland
[*]jerome.kasparian@unige.ch

ABSTRACT

This Supplementary Material gathers supplementary figures

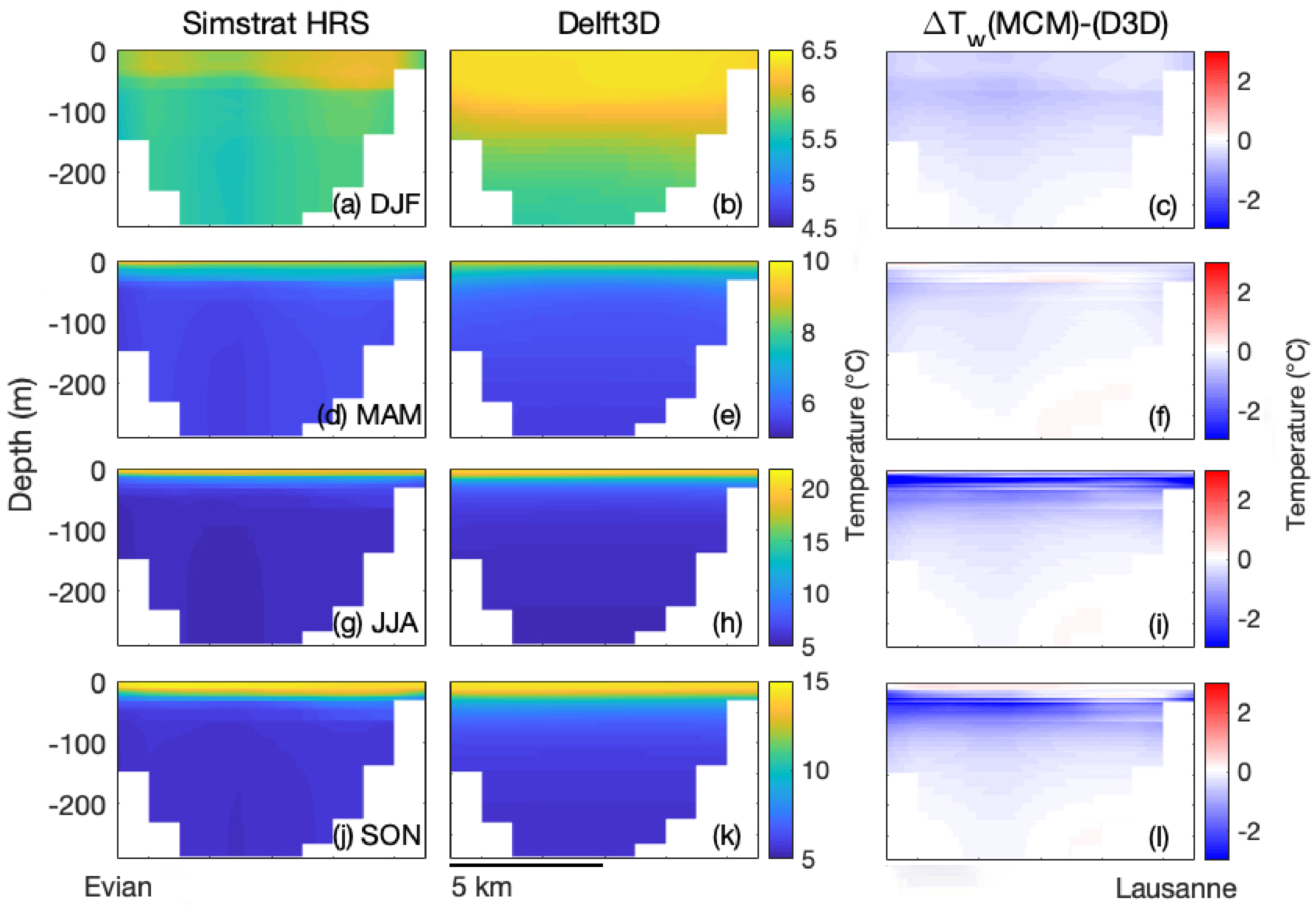

Figure S1. Season-averaged N–S transects (See Fig. 1) for MC-Simstrat (col 1), Delft3D (D3D, col 2), and the difference MC-Simstrat–Delft3D (col 3). (a–c) Winter (DJF), (d–f) Spring (MAM), (g–i) Summer (JJA), (j–l) Autumn (SON). See Figure S3 for monthly details.. Plots generated with Matlab[1]; Panels assembled with Adobe Illustrator[2]

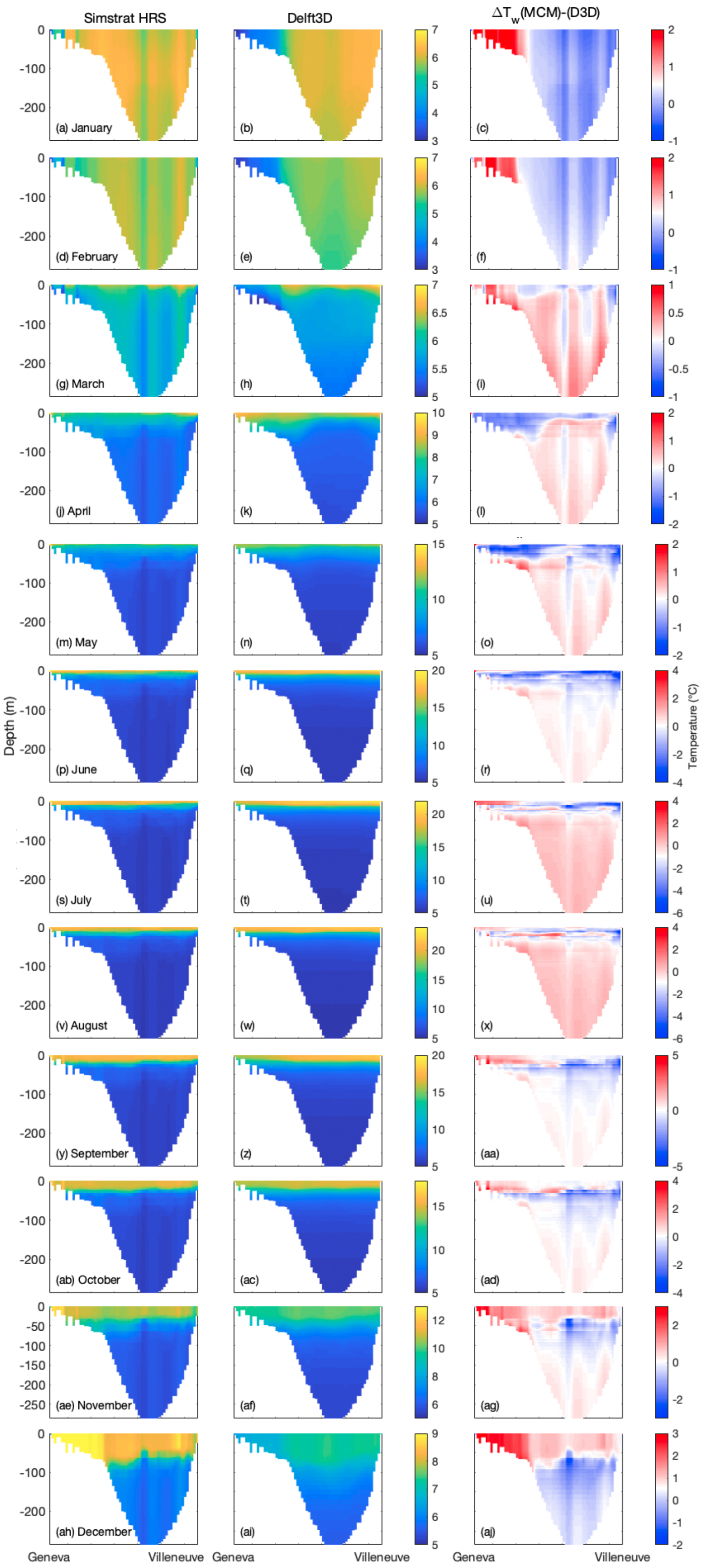

Figure S2. Monthly-averaged W–E transects (See Fig. 1) for MC-Simstrat (Column 1), Delft3D (Column 2), and the difference MC-Simstrat–Delft3D (Column 3). Months are sorted from January to December. Plots generated with Matlab[1]; Panels assembled with Adobe Illustrator[2]

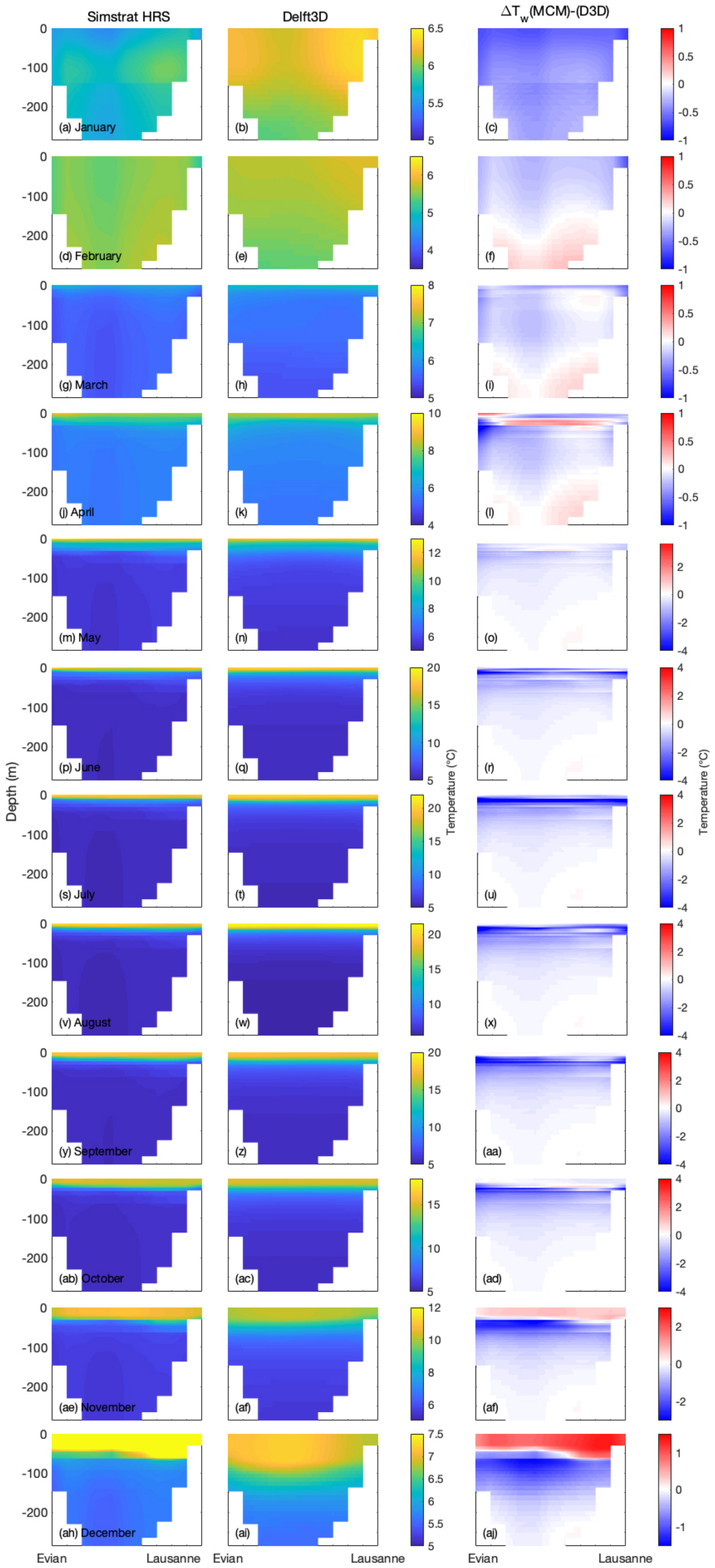

Figure S3. Monthly-averaged N–S transects (See Fig. 1) for MC-Simstrat (Column 1), Delft3D (Column 2), and the difference MC-Simstrat–Delft3D (Column 3). Months are sorted from January to December. Plots generated with Matlab[1]; Panels assembled with Adobe Illustrator[2]

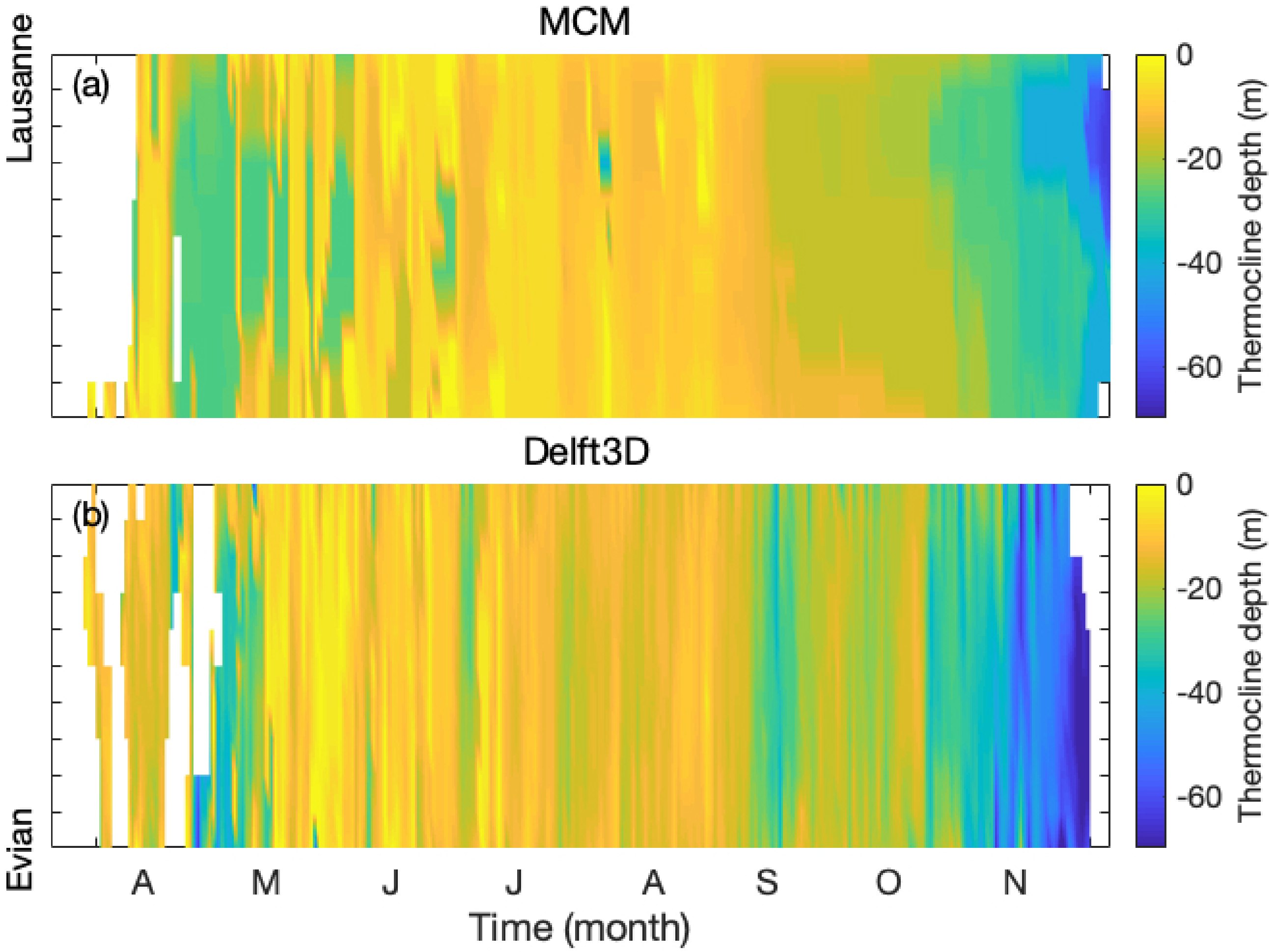

Figure S4. Temporal evolution of the thermocline depth over the the N–S transect, as simulated with (a) MC-Simstrat and (b) Delft3D. Plots generated with Matlab[1]; Panels assembled with Adobe Illustrator[2]

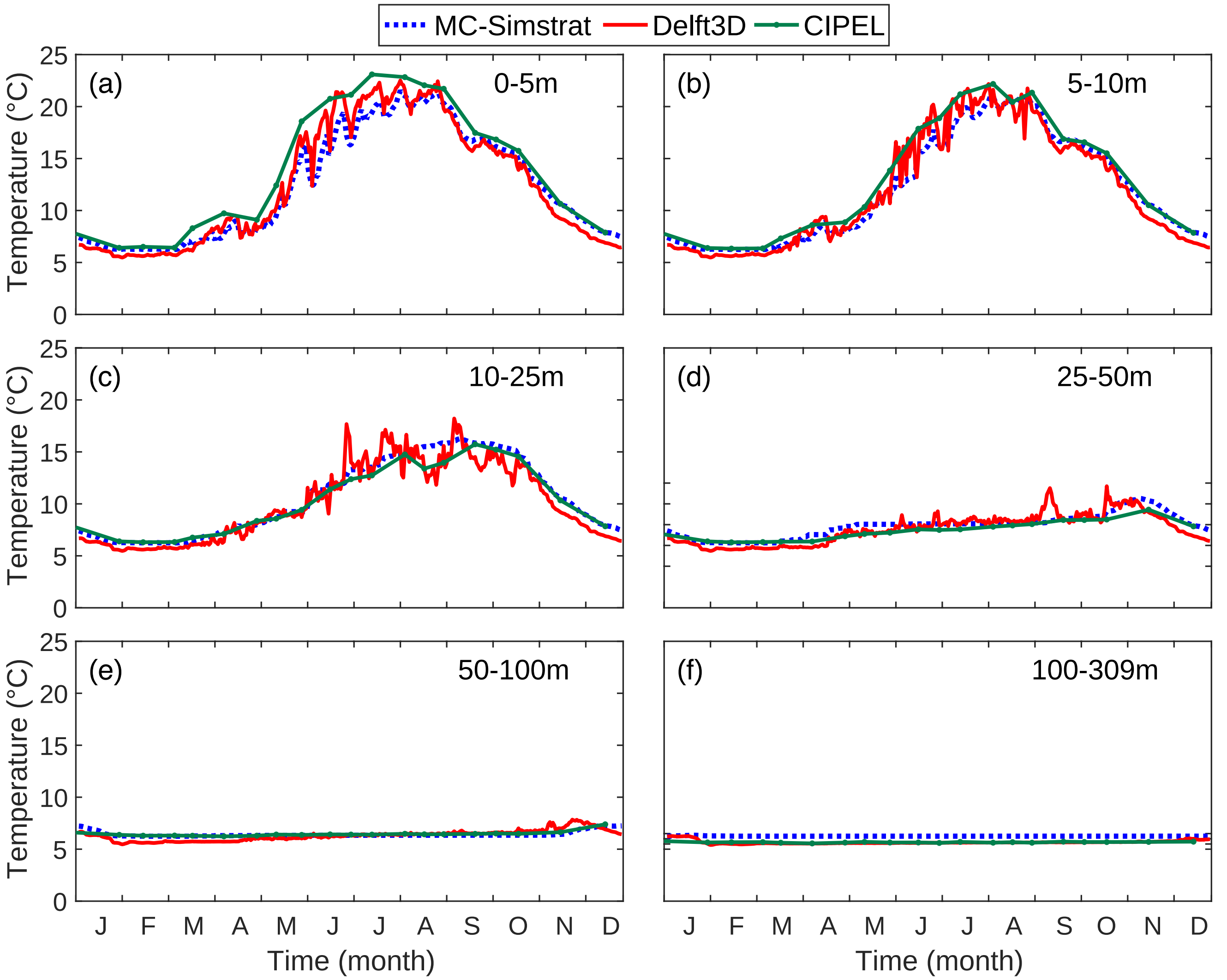

Figure S5. Evolution of the temperature simulated by MC-Simstrat (using the drag coefficient parametrization of Eq. (2) and Delft3D at 1100 UTC at station SHL2 at (a) the surface (0–5 m), (b) 5–10 m, (c) 10–25 m, (d) 25–50 m, (e) 50–100 m, (f) 100-309 m. Plots generated with Matlab[1]; Panels assembled with Adobe Illustrator[2]

References

1. Matlab r2018b, https://www.mathworks.com/products/matlab.html.

2. Adobe illustrator 2021, https://www.adobe.com/fr/products/illustrator.html.
